# Complete genome sequence of a commensal bacterium, *Hafnia alvei* CBA7124, isolated from human feces

**DOI:** 10.1186/s13099-017-0190-0

**Published:** 2017-07-27

**Authors:** Hye Seon Song, Joon Yong Kim, Yeon Bee Kim, Myeong Seon Jeong, Jisu Kang, Jin-Kyu Rhee, Joseph Kwon, Ju Suk Kim, Jong-Soon Choi, Hak-Jong Choi, Young-Do Nam, Seong Woon Roh

**Affiliations:** 1 Microbiology and Functionality Research Group, World Institute of Kimchi, Gwangju, 61755 Republic of Korea; 2 Department of Food Science and Engineering, Ewha Womans University, Seoul, 03760 Republic of Korea; 3 Chuncheon Center, Korea Basic Science Institute, Gangneung, Gangwon-do 24341 Republic of Korea; 4 Gut Microbiome Research Group, Korea Food Research Institute, Seongnam, 13539 Republic of Korea; 5 University of Science and Technology, Daejeon, 34113 Republic of Korea; 6 Biological Disaster Analysis Group, Korea Basic Science Institute, Daejeon, 34133 Republic of Korea

**Keywords:** *Hafnia alvei* CBA7124, Complete genome sequence, Gut microbiota, Comparative genomics

## Abstract

**Background:**

Members of the genus *Hafnia* have been isolated from the feces of mammals, birds, reptiles, and fish, as well as from soil, water, sewage, and foods. *Hafnia alvei* is an opportunistic pathogen that has been implicated in intestinal and extraintestinal infections in humans. However, its pathogenicity is still unclear. In this study, we isolated *H. alvei* from human feces and performed sequencing as well as comparative genomic analysis to better understand its pathogenicity.

**Results:**

The genome of *H. alvei* CBA7124 comprised a single circular chromosome with 4,585,298 bp and a GC content of 48.8%. The genome contained 25 rRNA genes (9 5S rRNA genes, 8 16S rRNA genes, and 8 23S rRNA genes), 88 tRNA genes, and 4043 protein-coding genes. Using comparative genomic analysis, the genome of this strain was found to have 72 strain-specific singletons. The genome also contained genes for antibiotic and antimicrobial resistance, as well as toxin–antitoxin systems.

**Conclusions:**

We revealed the complete genome sequence of the opportunistic gut pathogen, *H. alvei* CBA7124. We also performed comparative genomic analysis of the sequences in the genome of *H. alvei* CBA7124, and found that it contained strain-specific singletons, antibiotic resistance genes, and toxin–antitoxin systems. These results could improve our understanding of the pathogenicity and the mechanism behind the antibiotic resistance of *H. alvei* strains.

**Electronic supplementary material:**

The online version of this article (doi:10.1186/s13099-017-0190-0) contains supplementary material, which is available to authorized users.

## Background

*Hafnia alvei* was first identified by Moller in 1954. It belongs to the family *Enterobacteriaceae*, and was isolated from the feces of mammals, birds, reptiles, and fish, as well as from soil, water, sewage, and foods [2]. *H. alvei* is a Gram-negative, rod-shaped, and facultative anaerobic bacterium. It is an opportunistic pathogen, and has been implicated in intestinal and extraintestinal infections in humans [2]. In addition, several strains of *H. alvei* have been known to produce acyl lactones and form biofilms [3]. Biofilm formation is considered an important virulence factor involved in bacterial attachment and settlement [4]. However, the pathogenesis and mechanisms of action of *H. alvei* are still not clear [5]. So far, 11 strains of *H. alvei* have been sequenced, and only three genomes of them were completed.

In this study, we isolated the strain, *Hafnia alvei* CBA7124, from human feces, and performed sequencing and comparative genomic analysis with other *H. alvei* strains in order to understand its pathogenicity. The complete genome sequence of *H. alvei* CBA7124 would improve our understanding of different strains of opportunistic infectious pathogens.

## Methods

### Bacterial strain and DNA preparation

The strain *H. alvei* CBA7124 was isolated from a fecal sample of 66-year old Korean female from Geochang, Republic of Korea. The fecal sample was cultured in a brain heart infusion agar (BD) in anaerobic conditions at 37 °C for 24 h. The isolate was transferred at least thrice in the same conditions. The cell morphology of the strain was examined using a scanning electron microscope (SEM). The strain was then preserved in 20% (v/v) glycerol at −80 °C. The genomic DNA of the isolated strain was extracted using the QuickGene DNA tissue kit S (Kurabo, Japan) and purified using the MG Genomic DNA purification kit (Doctor Protein, Korea). The quality and concentration of the extracted DNA were determined using 1%-agarose gel electrophoresis and a NanoDrop spectrophotometer (Nanodrop Technologies, UK).

### Genome sequencing, assembly, and gene annotation

Whole genome sequencing was performed using Pacific Biosciences RS II (Pacific Biosciences, Menlo Park, USA) (Additional file 1: Table S1). A 20-kb sequencing library was constructed using SMRTbell™ Template Prep Kit and sequenced with P6 polymerase and C4 chemistry. The genome was assembled according to the protocol in the Hierarchical Genome Assembly Process version 2 with PacBio SMRT analysis version 2.3, and polishing was performed with Quiver. Identification of rRNA and tRNA genes was performed with the RNAmmer 1.21 [6] server and the tRNA scan-SE 1.21 [7], respectively. Functional genes were predicted and annotated using the SEED subsystems in the RAST server (rapid annotation using subsystem technology) [8, 9] and the COG (clusters of orthologous groups of proteins) databases [10]. The presence of CRISPRs was detected using the CRISPRfinder server [11]. PathogenFinder was used for predicting pathogenicity towards humans [12]. The ResFinder program was used to screen for antimicrobial resistance genes [13].

### Comparative genomic analysis

Comparative genomic analysis was performed on 11 *Hafnia alvei* strains, ATCC 29926, ATCC 13337^T^, DSM 30099, FB1, HUMV-5920, DSM 30098, LE8, GB001, FDAARGOS_158, bta3-1, and ATCC 51873. The orthologous average nucleotide identity (orthoANI) algorithm was used to measure the phylogenetic distances between these strains [14]. Pan-genome orthologous groups (POGs) were identified using the EzBioCloud Comparative Genomics Database (http://cg.ezbiocloud.net/). The heat map was clustered according to the presence or absence of genes [15].

### Quality assurance

Before the genome sequencing, the identity of the *H. alvei* CBA7124 strain was verified through 16S rRNA gene sequencing and cell morphology analysis (Additional file 1: Figure S1). In addition, the identity of the strain CBA7124 was confirmed through analysis of the 16S rRNA gene obtained after genome sequencing. In addition, we used the orthoANI values with the genome sequence of *H. alvei*.

## Results and discussion

### Genome characteristics

**Fig. 1:**
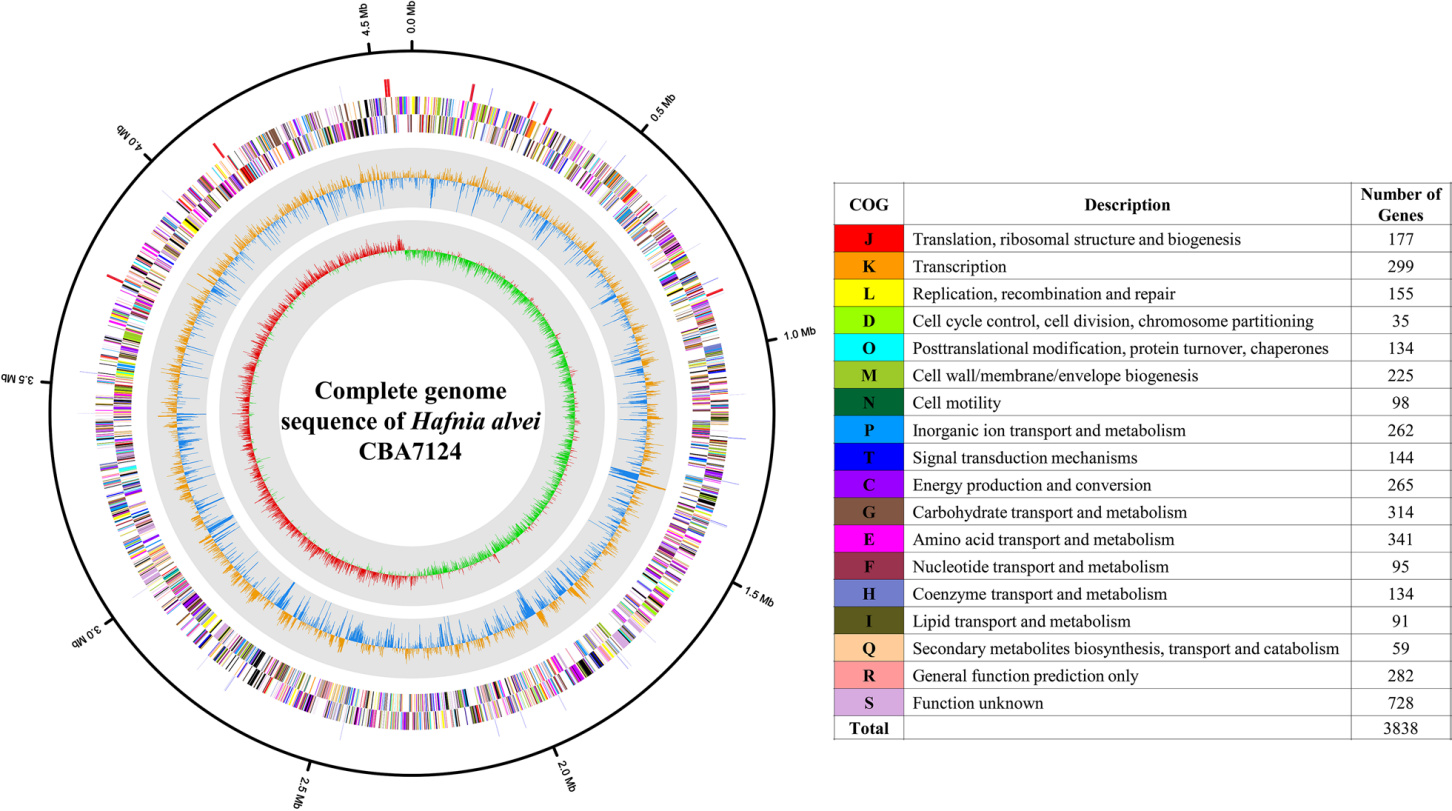
Circular genome map of *Hafnia alvei* CBA7124. From outer to inner rings, the individual *circles* indicate rRNAs and tRNAs, reverse CDSs, forward CDSs, GC skew, and GC ratio

The analysis of the whole genome sequence of *H. alvei* CBA7124 revealed a single circular chromosome with 4,585,298 bp, after quality control of 150,292 raw reads with an average read length of 5885 bp (Table 1). The genome coverage was found to be 168.69-fold and the GC content was 48.8%. The genome contained 25 rRNA genes (9 5S rRNA genes, 8 16S rRNA genes, and 8 23S rRNA genes) and 88 tRNA genes. Four confirmed CRISPRs (with at least three motifs and at least two exactly identical direct repeats) and four questionable CRISPRs (small CRISPRs or structures where the repeated motifs are not 100% identical) were found. The CRISPR-associated (Cas) proteins belong to the types I (Cas3), II (Cas1), IF (Csy1, Csy2, and Csy3 family), and IIB (Csy4 family), as confirmed from the SEED database. The strain had a 0.65% chance of being pathogenic, and was found to match with 28 pathogenic families. The genome contained 4043 protein-coding genes (CDSs) and 3838 genes were allotted to 18 COG functional categories. In the COG distribution, amino acid transport and metabolism (E; 341 ORFs), carbohydrate transport and metabolism (G; 314 ORFs), transcription (K; 299 ORFs), general function prediction only (R; 282 ORFs), and function unknown (S; 728 ORFs) were the major functional categories (Fig. 1). In the SEED subsystem distribution, carbohydrates (552 ORFs), amino acids and derivatives (430 ORFs), cofactors, vitamins, prosthetic groups, pigments (319 ORFs), and RNA metabolism (215 ORFs) were the abundant categories.

**Table 1:** Complete genome features of *Hafnia alvei* CBA7124

Attribute	Value
Topology	Crcular
Genome size (bp)	4,585,298
DNA G + C (%)	48.8
Genome coverage (fold)	168.69
Number of tRNA genes	88
Number of rRNA genes	25
Number of CDSs	4043
Genes assigned to COGs	3838
Confirmed CRISPRs	4

### Comparative genomic analysis

**Fig. 2:**
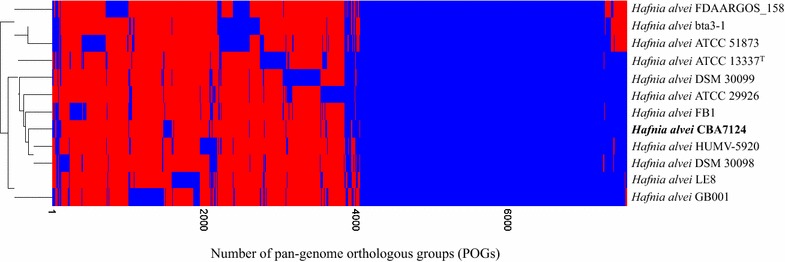
Heat map of strain CBA7124 with the related *Hafnia alvei* strains, constructed based on the presence or absence of POGs. The presence and absence of POGs are indicated by *blue* and *red*, respectively

The genome of the *H. alvei* CBA7124 strain was compared with those of 11 other *H. alvei* strains. The orthoANI values of strain CBA7124 with ATCC 13337^T^, ATCC 29926, DSM 30099, FB1, HUMV-5920, DSM 30098, LE8, GB001, FDAARGOS_158, bta3-1, and ATCC 51873 were 99.1, 99.0, 97.8, 97.7, 95.8, 95.7, 94.3, 92.9, 82.6, 82.5, and 82.5%, respectively, indicating that the strain CBA7124 was closely related to the *H. alvei* strains ATCC 13337^T^ and ATCC 29926 (Additional file 1: Figure S2). According to the heat map generated based on core pan-genome orthologous groups (POGs), the strain CBA7124 was clustered with *H. alvei* genomes of strains HUMV-5920 and DSM 30098, based on the presence or absence of genes (Fig. 2). Based on the POG comparison analysis of the 12 genomes, 72 strain-specific singletons, including “transposase for insertion sequence element IS200”, “protein SamB”, “protein RhsA”, and others, were identified in strain CBA7124 (Additional file 1: Table S2). The number of strain-specific POGs in the *H*. *alvei* genomes ranged from 72 to 354 (Additional file 1: Table S3). These results indicated that the genome of strain CBA7124 was separate from, but highly homologous to, that of other *H. alvei* genomes.

### Antibiotic and antimicrobial resistance genes

In the genome of strain CBA7124, 12 kinds of subsystems were found to be associated with the subcategory “resistance to antibiotics and toxic compounds” on the SEED database. These subsystems included 6 mdtABCD multidrug resistance clusters, 2 lysozyme inhibitors, 1 multiple antibiotic resistance (MAR) locus, 6 copper homeostasis, 2 bile hydrolysis, 6 cobalt-zinc-cadmium resistance, 3 multidrug resistance tripartite systems found in gram negative bacteria, 4 resistance to fluoroquinolones, 3 arsenic resistance, 7 copper homeostasis: copper tolerance, 3 beta-lactamase, and 10 multidrug resistance efflux pumps (Additional file 1: Table S4). In addition, the antimicrobial resistance gene, blaACC-3, was also found to be associated with the beta-lactam resistance AmpC-type gene from the ResFinder server.

### Toxin–antitoxin systems

Several toxin–antitoxin (TA) systems were annotated in the genome of *Hafnia alvei* for stabilization, based on the SEED database. We detected the TA systems of yefM/yoeB, ccdAB, parDE, and ygiUT in *H. alvei* CBA7124, which have been reported to inhibit replication by inhibiting DNA gyrase and translation. Among them, the antitoxin of yefM is involved in the formation of biofilms [16] and the ability of the biofilm-forming bacteria to withstand antibiotics; therefore, it has a significant impact on therapy and patient care [17]. In addition, the overproduction of the toxin of yoeB is known to inhibit the growth of *E*. *coli* [18, 19].

### Future directions

We described a genome sequence of *H. alvei*, a known opportunistic pathogen isolated from a Korean fecal sample. This genome was found to have strain-specific singletons through comparative genomic analysis with the other *H. alvei* strains. In addition, this genome contained antibiotic and antimicrobial resistance genes, toxin–antitoxin systems, and several Cas proteins against pathogen defence systems. This information provides new insights into the multidrug resistance, biofilm formation, and antibacterial activity of *H. alvei* for surviving in the intestinal environment. Furthermore, it can help us comprehend the pathogenesis and mechanisms of action of *H. alvei*. The data presented in this report provide important genetic information and a framework for further research. However, further in vivo studies are needed to characterize the pathogenicity of *H. alvei*.

## Additional file

**Additional file 1: Table S1.** Genome sequencing information for *Hafnia alvei* CBA7124. **Table S2.** Strain-specific singletons of *Hafnia alvei* CBA7124 based on the comparison of POGs (without 3 uncharacterized proteins and 54 hypothetical proteins). **Table S3.** Comparison of genome characteristics and strain-specific singletons in *Hafnia alvei* strains. **Table S4.** Antibiotic and antimicrobial resistance genes of *Hafnia alvei* CBA7124. **Figure S1.** Cell Morphology of *Hafnia alvei* CBA7124 viewed using SEM. Bar size = 1 μm. **Figure S2.** OrthoANI dendrogram of *Hafnia alvei* CBA7124 with other *H. alvei* genomes.

## Abbreviations

CDS: protein coding gene
COG: clusters of orthologous groups
POG: pan-genome orthologous group
orthoANI: orthologous average nucleotide identity
RAST: rapid annotation using subsystem technology
TA: toxin–antitoxin
