# Supplementary material for: Complete genome sequence of a commensal bacterium, Hafnia alvei CBA7124, isolated from human feces
Source: Gut Pathog. 2017 Jul 27;9:41. doi: 10.1186/s13099-017-0190-0 (PMC5530468; doi:10.1186/s13099-017-0190-0)

**Additional Files**

**Complete genome sequence of a commensal bacterium, *Hafnia* *alvei* CBA7124, isolated from human feces**

Hye Seon Song^1,2^, Joon Yong Kim^1^, Yeon Bee Kim^1,2^, Myeong Seon Jeong^3^, Jisu Kang^4,5^, Jin-Kyu Rhee^2^, Joseph Kwon^6^, Ju Suk Kim^5,6^, Jong-Soon Choi^6^, Hak-Jong Choi^1^, Young-Do Nam^4,5†^, and Seong Woon Roh^1^*^†^

^1^ Microbiology and Functionality Research Group, World Institute of Kimchi, Gwangju 61755, Republic of Korea

^2^ Department of Food Science and Engineering, Ewha Womans University, Seoul 03760, Republic of Korea

^3^ Chuncheon Center, Korea Basic Science Institute, Gangneung, Gangwon-do 24341, Republic of Korea

^4^ Gut Microbiome Research Group, Korea Food Research Institute, Seongnam 13539, Republic of Korea

^5^ University of Science and Technology, Daejeon 34113, Republic of Korea

^6^ Biological Disaster Analysis Group, Korea Basic Science Institute, Daejeon 34133, Republic of Korea

^†^ These authors contributed equally to this work

* Corresponding author:

S.W. Roh: Microbiology and Functionality Research Group, World Institute of Kimchi, Gwangju 61755, Republic of Korea. Telephone: +82 62 610 1778; Fax: +82 62 610 1853; E-mail: seong18@gmail.com

**Table S1.** Genome sequencing information for *Hafnia alvei* CBA7124.

| Property | Term |
| --- | --- |
| Finishing quality | Finished |
| Libraries used | PacBio SMRTbell™ library |
| Sequencing platforms | PacBio RS II sequencer |
| Assemblers | PacBio SMRT analysis 2.3 |
| Gene calling method | RAST ver. 2.0 |
| Number of SMRT cells | 1 |
| Number of reads | 150,292 |
| Average genome coverage | 168.69 x |
| Contigs no. | 1 |
| Chromosome length (bp) | 4,585,298 |
| Locus Tag | CBA7124 |
| Genbank ID | AP017469 |
| Genbank Date of Release | 01-Jun-16 |
| BIOPROJECT | PRJDB4840 |
| Source Material Identifier | CBA7124 |

**Table S2.** Strain-specific singletons of *Hafnia alvei* CBA7124 based on the comparison of POGs (without 3 uncharacterized proteins and 54 hypothetical proteins).

| Singleton | EggNog ID | Product |
| --- | --- | --- |
| 1 | S:COG0714 | Alkaline phosphatase |
| 2 | M:ENOG410XNNB | Cyclic di-GMP-binding protein |
| 3 | G:COG0477 | Protein TsgA like protein |
| 4 | O:ENOG4111ZA2 | Probable fimbrial chaperone LpfB |
| 5 | S:ENOG4111N65 | Chaperone protein FanE |
| 6 | M:COG3188 | Outer membrane usher protein HtrE |
| 7 | T:ENOG410XNMH | Diguanylate cyclase |
| 8 | L:COG0389 | Protein SamB |
| 9 | K:COG0789 | Uncharacterized HTH-type transcriptional regulator |
| 10 | M:COG3209 | Protein RhsA |
| 11 | T:COG0664 | Regulatory protein |
| 12 | L:COG1943 | Transposase for insertion sequence element IS200 |
| 13 | M:COG1462 | Curli production assembly/transport component CsgG |
| 14 | S:ENOG4112C48 | Lipopolysaccharide core biosynthesis protein RfaS |
| 15 | L:COG0210 | DNA helicase |

**Table S3.** Comparison of genome characteristics and strain-specific singletons in *Hafnia alvei* strains

|  | Species | No. of contigs | Genome size (bp) | DNA G+C content (%) | No. of CDSs | No. of rRNA genes | No. of tRNA genes | No. of singletons |
| --- | --- | --- | --- | --- | --- | --- | --- | --- |
| 1 | *Hafnia alvei* CBA7124 | 1 | 4,585,298 | 48.8 | 4,043 | 25 | 88 | 72 |
| 2 | *Hafnia alvei* ATCC 29926 | 64 | 4,742,049 | 48.7 | 4339 | 3 | 75 | 121 |
| 3 | *Hafnia alvei* ATCC 13337^T^ | 161 | 4,820,018 | 48.7 | 4485 | 4 | 64 | 199 |
| 4 | *Hafnia alvei* DSM 30099 | 53 | 4,804,570 | 48.7 | 4452 | 3 | 83 | 251 |
| 5 | *Hafnia alvei* FB1 | 1 | 4,712,721 | 49.0 | 4264 | 25 | 92 | 214 |
| 6 | *Hafnia alvei* HUMV-5920 | 2 | 4,630,439 | 48.7 | 4149 | 22 | 90 | 85 |
| 7 | *Hafnia alvei* DSM 30098 | 61 | 4,473,943 | 48.6 | 4038 | 4 | 74 | 338 |
| 8 | *Hafnia alvei* LE8 | 89 | 4,667,142 | 49.0 | 4276 | 4 | 74 | 251 |
| 9 | *Hafnia alvei* GB001 | 88 | 4,861,055 | 48.6 | 4478 | 3 | 68 | 145 |
| 10 | *Hafnia alvei* FDAARGOS_158 | 1 | 4,513,426 | 48.2 | 4132 | 25 | 87 | 236 |
| 11 | *Hafnia alvei* bta3-1 | 1 | 4,763,672 | 48.3 | 4243 | 21 | 72 | 103 |
| 12 | *Hafnia alvei* ATCC 51873 | 79 | 4,883,880 | 48.2 | 4514 | 4 | 65 | 354 |

**Table S4.** Antibiotic and antimicrobial resistance genes of *Hafnia alvei* CBA7124.

| Subsystem | Role |
| --- | --- |
| The mdtABCD multidrug resistance cluster | Multidrug transporter MdtC |
|  | Response regulator BaeR |
|  | Multidrug transporter MdtB |
|  | Probable RND efflux membrane fusion protein |
|  | Sensory histidine kinase BaeS |
|  | Multidrug transporter MdtD |
| Lysozyme inhibitors | Membrane-bound lysozyme inhibitor of c-type lysozyme |
|  | Inhibitor of vertebrate lysozyme precursor |
| Multiple Antibiotic Resistance MAR locus | Multiple antibiotic resistance protein MarC |
| Copper homeostasis | Cytochrome c heme lyase subunit CcmF |
|  | Cytochrome c heme lyase subunit CcmH |
|  | Copper-translocating P-type ATPase (EC 3.6.3.4) |
|  | Copper resistance protein CopC |
|  | Blue copper oxidase CueO precursor |
|  | Copper resistance protein D |
| Bile hydrolysis | DamX, an inner membrane protein involved in bile resistance |
|  | Choloylglycine hydrolase (EC 3.5.1.24) |
| Cobalt-zinc-cadmium resistance | Heavy metal RND efflux outer membrane protein, CzcC family |
|  | Cobalt-zinc-cadmium resistance protein |
|  | Cobalt/zinc/cadmium efflux RND transporter, membrane fusion protein, CzcB family |
|  | Cobalt-zinc-cadmium resistance protein CzcA |
|  | Zinc transporter ZitB |
|  | Cation efflux system protein CusA |
| Multidrug Resistance, Tripartite Systems Found in Gram Negative Bacteria | Outer membrane component of tripartite multidrug resistance system |
|  | Membrane fusion component of tripartite multidrug resistance system |
|  | Inner membrane component of tripartite multidrug resistance system |
| Resistance to fluoroquinolones | DNA gyrase subunit B (EC 5.99.1.3) |
|  | DNA gyrase subunit A (EC 5.99.1.3) |
|  | Topoisomerase IV subunit B (EC 5.99.1.-) |
|  | Topoisomerase IV subunit A (EC 5.99.1.-) |
| Arsenic resistance | Arsenical resistance operon repressor |
|  | Arsenic efflux pump protein |
|  | Arsenate reductase (EC 1.20.4.1) |
| Copper homeostasis: copper tolerance | Secreted protein, suppressor for copper-sensitivity ScsC |
|  | Membrane protein, suppressor for copper-sensitivity ScsB |
|  | Membrane protein, suppressor for copper-sensitivity ScsD |
|  | Copper homeostasis protein CutE |
|  | Periplasmic divalent cation tolerance protein CutA |
|  | Magnesium and cobalt efflux protein CorC |
|  | Copper homeostasis protein CutF precursor |
| Beta-lactamase | Beta-lactamase class C and other penicillin binding proteins |
|  | Beta-lactamase (EC 3.5.2.6) |
|  | Metal-dependent hydrolases of the beta-lactamase superfamily I |
| Multidrug Resistance Efflux Pumps | RND efflux system, outer membrane lipoprotein, NodT family |
|  | RND efflux system, outer membrane lipoprotein CmeC |
|  | Multi antimicrobial extrusion protein (Na(+)/drug antiporter), MATE family of MDR efflux pumps |
|  | Transcription repressor of multidrug efflux pump acrAB operon, TetR (AcrR) family |
|  | RND efflux system, inner membrane transporter CmeB |
|  | Multidrug-efflux transporter, major facilitator superfamily (MFS) (TC 2.A.1) |
|  | Macrolide export ATP-binding/permease protein MacB (EC 3.6.3.-) |
|  | Membrane fusion protein of RND family multidrug efflux pump |
|  | Macrolide-specific efflux protein MacA |
|  | Type I secretion outer membrane protein, TolC precursor |

**Figure S1.** Cell Morphology of *Hafnia alvei* CBA7124 viewed using SEM. Bar size = 1 μm.


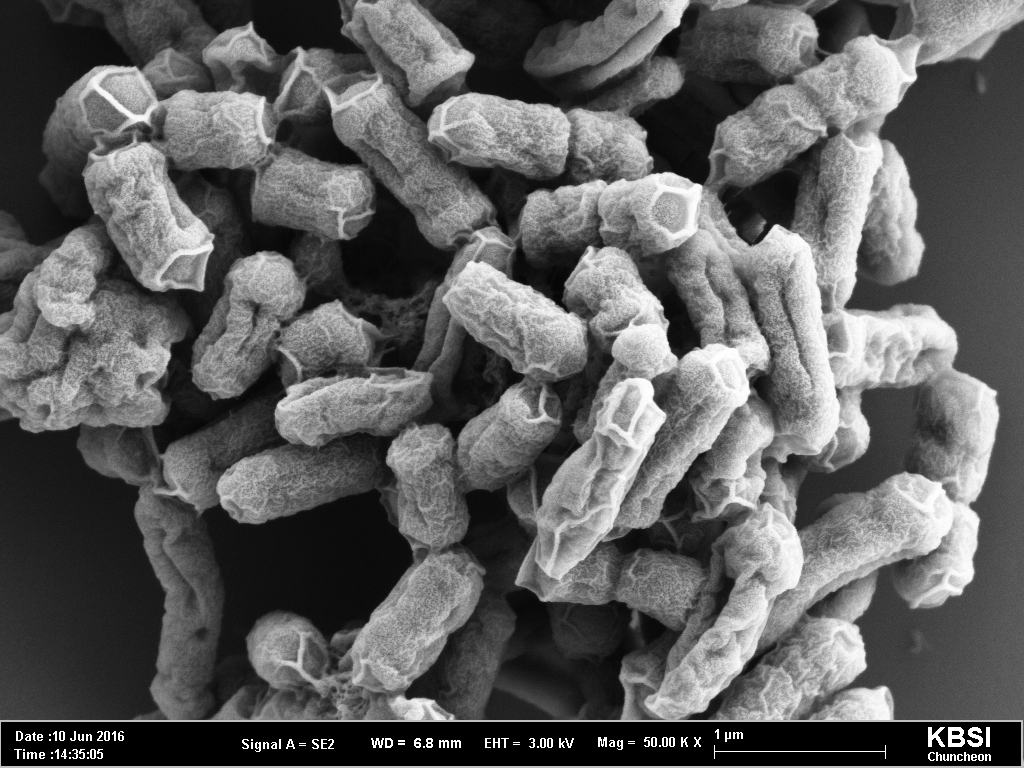


**Figure S2.** OrthoANI dendrogram of *Hafnia alvei* CBA7124 with other *H. alvei* genomes.


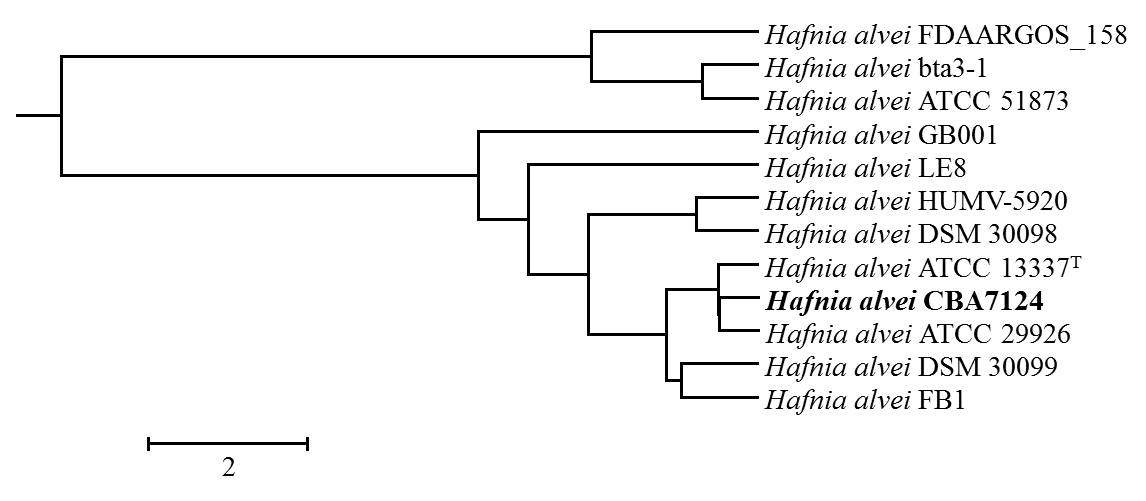

Supplement: Supplementary file 1 — Additional file 1: Table S1. Genome sequencing information for Hafnia alvei CBA7124. Table S2. Strain-specific singletons of Hafnia alvei CBA7124 based on the comparison of POGs (without 3 uncharacterized proteins and 54 hypothetical proteins). Table S3. Comparison of genome characteristics and strain-specific singletons in Hafnia alvei strains. Table S4. Antibiotic and antimicrobial resistance genes of Hafnia alvei CBA7124. Figure S1. Cell Morphology of Hafnia alvei CBA7124 viewed using SEM. Bar size = 1 μm. Figure S2. OrthoANI dendrogram of Hafnia alvei CBA7124 with other H. alvei genomes. [file 13099_2017_190_MOESM1_ESM.docx]
